# The importance of illness duration, age at diagnosis and the year of diagnosis for labour participation chances of people with chronic illness: results of a nationwide panel-study in the Netherlands

**DOI:** 10.1186/1471-2458-13-803

**Published:** 2013-09-04

**Authors:** Mieke Rijken, Peter Spreeuwenberg, Joop Schippers, Peter P Groenewegen

**Affiliations:** 1 NIVEL (Netherlands institute for health services research), P.O. Box 1568, 3500 BN, Utrecht, The Netherlands; 2 Faculty of Law, Economics and Governance, Utrecht University, P.O. Box 80125, 3508 TC, Utrecht, The Netherlands; 3 Faculty of Geoscience, Faculty of Social Science, Utrecht University, P.O. Box 80125, 3508 TC, Utrecht, The Netherlands

**Keywords:** Chronic illness, Labour participation, Employment, Age, Illness duration

## Abstract

**Background:**

Compared to participation rates among general populations, participation of people with chronic illness in the labour market lags behind. This is undesirable, both from the perspective of individuals’ well-being as from a macro-economic perspective for western countries where concerns exist about labour supply and sustainability of social security in the near future. To help develop successful policy measures to prevent early drop-out and support reintegration, we aimed to gain insight into the role of three age related characteristics that may relate to labour participation chances of people with chronic illness: the duration of their illness, how old they were when the chronic disease was diagnosed and the historical year in which the diagnosis was established.

**Methods:**

We analyzed data of one (first) measurement of several cohorts of people diagnosed with a somatic chronic disease, who (had) participated in the Dutch ‘National Panel of people with Chronic illness or Disability’ since 1998 (N = 4634 in total). Multi-level logistic regression analyses were conducted to estimate random effects of the age at diagnosis and the year of diagnosis and fixed effects of illness duration on labour participation, while correcting for the effects of socio-demographic and disease characteristics and socio-economic indicators.

**Results:**

A significant part of the variation in labour participation among people with chronic illness relates to the age they had when they were diagnosed. Furthermore, a longer illness duration is significantly associated with a lower chance of being economically active. This is more the case for men than for women. Labour participation of cancer survivors depends on the phase of the illness they find themselves in. No evidence was found that the year in which the diagnosis was established matters for employment chances later in life.

**Conclusion:**

Age at diagnosis and illness duration relate to chronically ill people’s chances to participate in the labour market, but how and how strong they relate to labour participation depend on gender and the type of chronic disease at stake. Prospective studies are needed to assess illness trajectories of specific diagnostic groups along with the development of their school and work careers.

## Background

In view of the ageing of their populations, western governments are concerned whether there will be enough workers available in the near future to match the demand for labour and to keep social security affordable [1]. Policy measures have been taken to stimulate labour participation among target groups who are now underrepresented at the labour market and to prevent early drop-out. In the Netherlands, several laws have been adopted in the last decade to impose employers to put more effort to prevent prolonged sick-leave and occupational disability of their employees as well as to stimulate economically inactive people with chronic illness or functional disabilities to join the labour market. However, although decreases have been observed in sick-leave and new disability insurance beneficiaries, labour participation rates among people with long-term health impairments have not increased [2,3]. Labour participation among people with chronic illness or functional disabilities in the Netherlands is still low. In 2010, the percentages of people with somatic chronic illness or disabilities found to have a paid job for at least 12 hours a week were 48% (15 to 39 years), 38% (40 to 54 years) and 16% (55 to 64 years) [4]. In the total Dutch population, labour participation rates in 2010 were 66% (15–39 years) and 80% (40–54 years), then dropped to 49% among people aged 55 and older. *[Data provided on request by Statistics Netherlands in April 2011; data from the Survey Working Population 2010.]* Similar patterns among general populations have been found for all European Union countries together and for Europe as a whole, whereas for the United States the decline after 55 is less sharp [5].

Many studies have investigated the relationship between health and labour participation, showing in general that poor health is associated with non-participation, and that chronic illness is more common among persons not having a paid job than among employed persons [e.g. [6-14]. These results can be explained from either the causation hypothesis (non-participation causes ill health) and/or the health selection hypothesis (poor health decreases employment chances and effectuates early exit from labour force) [15]. Several authors found that the relationship between poor health and exit from paid employment is not straightforward, but influenced by personal characteristics such as gender and education, lifestyle and working conditions [e.g. [7,9,10]. In this paper we further explore the role of chronic illness in the selection process into labour participation. We wish to shed light on some characteristics related to ageing with a chronic illness that may explain labour participation variation among people with chronic illness and that may be of interest for the development of successful policy measures to support their participation: 1. illness duration, 2. the age at which a chronic disease had been diagnosed, and 3. the historical time period in which the diagnosis was established.

### Illness duration

Knowing that chronic diseases can vary substantially regarding their course and burden, their influence on labour participation may be different for different diagnostic groups (people with diabetes, arthritis, cancer, et cetera), but also at different stages of the illness process. For instance, diabetic patients may have felt unwell and tired before diagnosis, but they may recover quickly and experience more energy once they are treated appropriately. Nevertheless, complications may occur in the long run and these can affect activity levels, resulting in sick-leave and occupational disability in the end. The course of arthritis is completely different, usually starting with mild symptoms, which progress steadily over time and ultimately result in severe invalidation. Finally, most cancer types are characterized by a first phase of acute crisis, which -when initial treatment has been successful- is followed by a phase of rehabilitation and recovery, which at its turn often results in a chronic phase where the disease is in remission but energy levels may remain sub-optimal for years due to severe treatment. Based on an analysis of 12 studies published between 1985 and 1999, Spelten and colleagues conclude that on average 65 percent of all cancer patients who were employed before the diagnosis of cancer return to work. A closer look reveals that 76 percent of those with an illness duration (years post-diagnosis) of two years at a maximum return to work. Among cancer patients with an illness duration between two and five years, 63 percent of those originally employed had returned to work, and among those diagnosed more than five years ago this percentage was 67 [16].

### Age at diagnosis

Besides the fact that different chronic diseases have different courses that may interfere with the ability to perform (paid) work, it is also likely that their impact on labour participation depends on the age or phase of life in which they arise. For instance, being diagnosed with a chronic disease during childhood may have had a negative impact on one’s school career. This can be understood from the health selection hypothesis and more specifically the process of social stunting, which posits that poor health, particularly during critical and sensitive periods of childhood and adolescence, may limit an individual’s early accumulation of human capital [17]. A systematic review revealed evidence that children with diabetes missed more school than other children [18]. Some indication was found that childhood-onset diabetes is associated with disadvantage in employment. More specifically, an early British study showed that people who were diagnosed with diabetes during early childhood were more likely to be employed than those who developed this condition during adolescence [19]. Adolescents and young adults with inflammatory bowel disease or chronic liver disease were more often absent from school or study due to illness and less often employed than age and gender matched healthy controls [20]. Young adults who had suffered from asthma during childhood or adolescence were not less often employed than non-asthmatics, but reported slightly more limitations in their vocational and working careers [21]. Case and colleagues examined data from the 1958 National Child Development Study in Great Britain (all children born in Scotland, England and Wales in the week of March 3, 1958 from birth through to age 42). They found that for every additional chronic condition at age 16, there was a four percent point reduction in the probability of employment at age 33, and a five percent point reduction in the probability of employment at age 42 [22]. Other studies have also demonstrated adverse effects of poor childhood health on socioeconomic position including labour participation [17,23,24]. However, the studies mentioned here all focused on the negative impact of poor health or the presence of chronic illness during childhood or adolescence, but it is also likely that being diagnosed with a chronic disease at the age of 30 will have a more destructive influence on one’s career than being diagnosed with the same disease at the age of 50, since the accumulation of human capital will normally continue during an individual’s work career and will thus be affected more when a chronic disease manifests itself at age 30 than at age 50.

### Historical period

The third factor that may relate to the likelihood of performing paid work when chronically ill is the historical period in which the diagnosis had been established. Two types of developments may be relevant in this respect: medical technical developments, and developments in the state of the economy and social security. Medical technical advances during the last 50 years have gradually led to better treatment options in many chronic diseases. For instance, being diagnosed with insulin dependent diabetes mellitus in the seventies was a completely different experience than being diagnosed with the same disease nowadays. In the mid seventies, patients who did not produce enough insulin themselves had to follow a strict diet and usually had to inject themselves with (animal-source) insulin that asked for a very fixed arrangement of their days. Insulin products of today allow patients to be much more flexible in their movements and activities, including the performance of paid work. Another example is the treatment of cancer. Due to improved treatments and earlier diagnosis, survival rates of cancer patients have increased impressively. In the USA, the five-year survival rate (for all cancer sites combined) raised from 50% in 1974 to 63% in 2000 [25]. In the Netherlands, the five-year survival rate (all cancer sites combined) among male patients raised from 30 percent in the seventies to 52 percent over the period 1999–2008, and among female patients from 45 to 61 percent over the same period [26,27]. The largest improvement in five-year survival has occurred among cancer patients aged 15–34 years: from 55 percent in the seventies to about 80 percent in 2001 [26].

Whether or not people with chronic illness are economically active is also influenced by the state of the economy and the social security system in a country [28]. Today’s economic situation and social security system will play a role as determinants of current labour participation, but also economic factors and social security at the time of diagnosis since these may have influenced the decision to remain (or, in case of young chronically ill, to become) active in the labour market or to stop working.

A high unemployment rate in a country at the time of diagnosis will negatively affect chronically ill people’s chances to remain employed or to (re)integrate in the labour market. This is due to the lower employment chances in general (also for healthy people), but it may be more distinct for people with chronic illness. For instance, when there is excess supply in the labour market, employers can be very selective when recruiting new employees, and companies who have to downsize may try to release chronically ill workers who they might consider less productive and less reliable than healthy workers. On the other hand, Schuring and colleagues demonstrated that higher national unemployment rates diminish the negative relationship between poor health and employment [10]. This suggests that in times of high unemployment other factors, besides health, may be selective as well. A country’s wealth (measured as its national income or Gross Domestic Product) may play an additional role, because it relates to the financial resources a country can spend on social security. In periods of high and growing wealth, welfare benefits will usually be generous. Some critics state that generous welfare benefits discourage economic activity among vulnerable groups such as people with chronic illness or disability, whereas others view such benefits as a social investment that enlarges people’s employment chances [29]. Van der Wel and colleagues demonstrated that in European countries with a high level of welfare generosity (in 2005), the presence of a disabling chronic illness had less negative effect on employment chances than in countries that spent less money to welfare [29]. Finally, disability insurance policy at the time of diagnosis may also influence (previous and current) labour participation. In times when broad eligibility criteria are used and disability benefits are relatively high, more people will apply for disability benefits and less will remain active in the labour market than in times of restricted entry and/or lower benefits [30].

### Hypotheses

Based on these considerations, we formulated the following hypotheses:

1. Illness duration: in general we expect that the longer people are chronically ill, the less likely it is that they participate in the labour market. This linear relationship is particularly expected for people diagnosed with a chronic disease with a usually progressive course (e.g. several musculoskeletal and neurological diseases). For people suffering from diseases that manifest themselves by an acute situation (e.g. myocardial infarction, several cancer types) the expected pattern is that of a low participation rate during the first year after diagnosis, then increasing participation in the next years, eventually followed by lower participation rates when the medical situation becomes more complex.

2. Age at diagnosis: we expect people who had become chronically ill at a younger age to be less likely to participate in the labour market, because of the larger negative impact the disease could have had at younger age on the development of a career.

3. Year of diagnosis: we expect an earlier year of diagnosis to be associated with a lower chance of current labour participation. First, because the year of diagnosis (as well as illness duration) relates to a person’s age and old age generally decreases the chance of being employed. Second, we expect people diagnosed in later years to be more often employed, because they may have benefited more from the development of better treatment options for many chronic diseases during the last decades. And third, we expect people diagnosed in later years to be more often employed because of the Dutch policy measures of recent years: a more restrictive disability insurance policy and a strong emphasis on (re)integration [31,32]. Again, the relationship between the year of diagnosis and labour participation may not be fully linear, because people with some chronic diseases may have benefited more from medical advances than people with other diseases, and disability insurance policy may have affected people with some chronic conditions more than others.

## Methods

This study formed part of a nationwide panel-study on the consequences of chronic illness as perceived by chronically ill people [33], called ‘National Panel of people with Chronic illness or Disability’ (NPCD). NPCD is registered with the Dutch Data Protection Authority. All data are collected and handled according to the privacy protection guidelines of the Authority. Data from the NPCD database are available on request from the project leader (MR), provided that the request meets the conditions^a^ for use as evaluated by the steering committee^b^ of the panel-study.

### Sample

Panel members were recruited from general practices (national random samples) [34] in 1998, 2001, 2005, 2006, 2007, 2008 and 2009. General practices can be considered the central sources of medical information, since general practitioners keep lifelong files of their patients. Inclusion criterion was a diagnosis of a somatic incurable disease. Exclusion criteria were: age < 15 years, being institutionalized, unaware of diagnosis, life expectancy < 6 months according to the GP, and insufficient mastery of the Dutch language. Eligible patients who agreed to participate filled in self-report questionnaires twice a year, for a maximum of four years. In this study we used data of only the first measurement of each cohort and confined the sample to patients aged 15 to 64. This resulted in a sample of 4634 unique persons (1998: 1138, 2001: 966, 2005: 967, 2006: 172, 2007: 353, 2008: 341 and 2009: 697), recruited from 181 general practices.

### Data

*Labour participation* was assessed by a self-report question about the social situation of the patient at that moment. Respondents who reported to work as an employee and/or to be self-employed were considered as participants in the labour market.^c^

*Disease type* was classified based on the (first) chronic disease patients had been diagnosed with [35] (as registered by the GP): cardiovascular, cancer, diabetes, asthma/COPD, musculoskeletal, neurological, digestive, and other. The *number of chronic diseases* registered by the GP had four values: one, two, three, and four or more chronic diseases. The *severity of the chronic condition* was assessed by the GP, based on three items (progressively deteriorating course, experience of pain and level of physical disability) rated on three-point scales: to a less extent (1), average (2), to a greater extent (3). Scale scores could range from 3 to 9 (Cronbach’s alpha .67).

*Year of diagnosis* (derived from the patient’s GP file) was a continuous variable that could range from 1935 until 2009. *Age at diagnosis* was computed by subtracting the year of birth (derived from the patient’s GP file and checked by the self-reported birth year) from the year in which the (first) chronic disease had been diagnosed, thus ranging from 0 to 64. *Illness duration* was constructed by subtracting the year in which the (first) chronic disease had been diagnosed from the year of measurement, resulting in a continuous variable ranging from 0 to 64.

*Socio-demographic characteristics* included were age, gender, ethnicity, education level, marital status, urbanisation level and country region of the place of residence. The variable *ethnicity* was based on the self-reported country of birth of the panel member, his/her father and mother and constructed in accordance with the construction rules of Statistics Netherlands [36], resulting in three groups: native, western non-native, and non-western inhabitants. *Education level* was based on the self-reported highest level of education accomplished by the respondent, ranging from primary school (1) to university (6). *Marital status* was self-reported and treated as a dichotomous variable: married/cohabiting versus single, including widowed and divorced. *Urbanisation level* was based on the four-digit postal code of the residential address of the panel member and ranged from most urban (1) to not urban (5). *Country region* (also derived from the four-digit postal code) had four options: north, east, west and south.

Based on national registration data [37,38], we constructed two variables as indicators of the economic situation in the year of diagnosis: *percentage of registered unemployment* and *annual rate of volume change in per capita Gross Domestic Product*. Panel members were assigned values for these variables based on their diagnostic year. Similarly we constructed a variable *disability insurance policy*. For this purpose we studied the Dutch policy on disability insurance from 1935 till 2009 [30,39,40]. We constructed a categorical variable with nine values: 1. years ≤ 1966, 2. 1967–1984, 3. 1985–1986, 4. 1987–1992, 5. 1993–2001, 6. 2002–2003, 7. 2004, 8. 2005, 9. 2006–2009. The border years correspond with important changes in the disability insurance policy in the Netherlands.^d^

It was also important to take into account the year in which the respondents filled in the questionnaire, because the economic situation in a specific year as well as the social security system or certain (political, natural) events might impact on labour participation in that year. Since we had a very limited number of (arbitrary) observation years and we only wanted to control for differences due to contextual factors, we included the *year of observation* as dummy variables in the analyses.

### Statistical analysis

Descriptive statistics were computed to provide information on sample characteristics and labour participation. The relationship between age and labour participation was assessed by (single-level) logistic regression analyses providing odds ratios of age for the total sample and the eight diagnostic groups separately. Pearson correlation coefficients were computed to assess the strength of the association between the number of years post-diagnosis (illness duration) on the one hand and severity of the disease(s) and the number of chronic diseases on the other.

To gain insight into the relationships of the three age related illness characteristics with labour participation, we conducted multi-level logistic regression analyses. Inspired by Hierarchical Age-Period-Cohort models [41-45], we specified a model assessing random effects of ‘age at diagnosis’ and ‘year of diagnosis’ (treating these variables as contexts shared by individuals rather than attributes of individuals) and fixed effects of illness duration (all separately for men and women). The model was cross-classified with respondents nested in both ‘age at diagnosis’ cohorts and ‘year of diagnosis’ periods. To account for potential non-linear effects of illness duration, it was included as three variables: illness duration in years, in years squared, and in years cubed. First, a model (0) specifying the random effects of age at diagnosis and the year of diagnosis was assessed, which also included gender and observation year (as a correction) in the fixed part. Next, a model (1) was assessed specifying the random effects as well as the fixed effects of gender and illness duration, corrected for the observation year, education level, marital status, region and urbanisation level of the place of residence of the panel members. Age was not included, since we aimed to unravel the effect of age on labour participation by including the respondent’s age at diagnosis and his/her illness duration. Ethnicity could not be included because of too little variation (see Results) and missing data (see Table 1). In the last model (2), the type and number of chronic diseases as well as the socio-economic indicators from the time of diagnosis were added to the fixed part.

**Table 1:** Socio-demographic characteristics of the sample (N = 4634)

	**N**	**n**	**%**	**Mean**	**Sd**
Gender	4634				
Male		1929	41.6		
Female		2705	58.4		
Age (in years)	4634			49.25	11.55
Ethnicity	3478^1^				
Native		3182	91.5		
Western non-native		219	6.3		
Non-western		77	2.2		
Education level	4503				
Primary school		400	8.9		
Low / preparatory Vocational education		1265	28.1		
Intermediate general education		795	17.7		
Advanced general or intermediate vocational education		1197	26.6		
High vocational education		655	14.5		
University		191	4.2		
Marital status	4622				
Single (incl. widowed, divorced)		940	20.3		
Married / cohabited		3682	79.7		
Urbanisation level	4634				
1: Most urban		550	11.9		
2: │		1052	22.7		
3: │		956	20.6		
4: │		1282	27.7		
5: Not urban		794	17.1		
Region of The Netherlands	4634				
North		396	8.5		
East		1415	30.5		
West		1843	39.8		
South		980	21.1		

^1^Information on ethnicity is missing for all cases joining the panel in 1998, because it was not included in the questionnaire they filled in at that time.

To provide disease-specific information, we conducted another multi-level logistic regression analysis with the variables age at diagnosis, year of diagnosis and illness duration separately specified for each diagnostic group. This model also included (in the fixed part) the year of observation, gender, education level, marital status, region and urbanisation level of the place of residence of the panel members, to correct for their effects. We wish to emphasize that the term ‘effect’ should not be interpreted as indicating causality; it is strictly used here in a technical (statistical) way.

## Results

### Sample characteristics

Sample characteristics are presented in Tables 1 and 2. The mean age at the time of diagnosis was 40 years, but the standard deviation (14.33) indicates a wide range. Participants were on average 10 years post-diagnosis when they reported their employment status. Significant positive associations were found between the number of years post-diagnosis (illness duration) and the total number of chronic diseases diagnosed (Pearson’s r = .19, P < .001), respectively the severity of the chronic condition as assessed by the GP (Pearson’s r = .05, P < .001).

**Table 2:** Illness characteristics of the sample (N = 4634)

		**N**	**n**	**%**	**Mean**	**Sd**
Type of chronic disease (index disease^1^)		4634				
	Cardiovascular		519	11.2		
	Cancer		196	4.2		
	Diabetes		576	12.4		
	Asthma / COPD		1080	23.3		
	Musculoskeletal		712	15.4		
	Neurological		441	9.5		
	Digestive		238	5.1		
	Other		872	18.8		
Year of diagnosis		4486				
	1935 – 1960		57	1.3		
	1960 – 1969		59	1.3		
	1970 – 1979		255	5.7		
	1980 – 1989		674	15.1		
	1990 – 1999		2057	45.9		
	2000 – 2009		1384	30.9		
Age at time of diagnosis		4486			40.19	14.33
	0 – 12 years		223	5.0		
	13 – 18 years		193	4.3		
	19 – 25 years		314	7.0		
	26 – 35 years		764	17.0		
	36 – 45 years		1070	23.9		
	46 – 55 years		1340	29.9		
	56 – 64 years		582	13.0		
Years post-diagnosis		4486			9.68	9.23
	Less than one year		172	3.8		
	One to two years		396	8.8		
	Two to five years		1074	23.9		
	Five to ten years		1216	27.1		
	Ten to 15 years		737	16.4		
	15 to 25 years		571	12.7		
	25 years or longer		320	7.1		
Total number of chronic diseases diagnosed		4634			1.36	0.66
	One		3392	73.2		
	Two		910	19.6		
	Three		254	5.5		
	Four or more		78	1.7		
Disease(s) severity as assessed by GP (range 3–9)		4571			4.83	1.68

^1^The index disease is the chronic disease that had been diagnosed first, in case of more than one chronic disease.

### Labour participation and age

Of the total sample, 48.5 percent had a paid job at the time of measurement. This percentage varied from 33.7 among cancer patients to 58.7 among patients diagnosed with asthma/COPD. The odds ratio of age on having paid work was 0.94 (P < .001), indicating a strong negative relationship between age and labour participation. The odds ratios of age for different diagnostic groups varied between 0.89 (cardiovascular disease) and 0.97 (asthma/COPD, neurological disease), and all of them were highly significant (P < .001).

### Effects of age at diagnosis, year of diagnosis and illness duration

**Figure 1:**
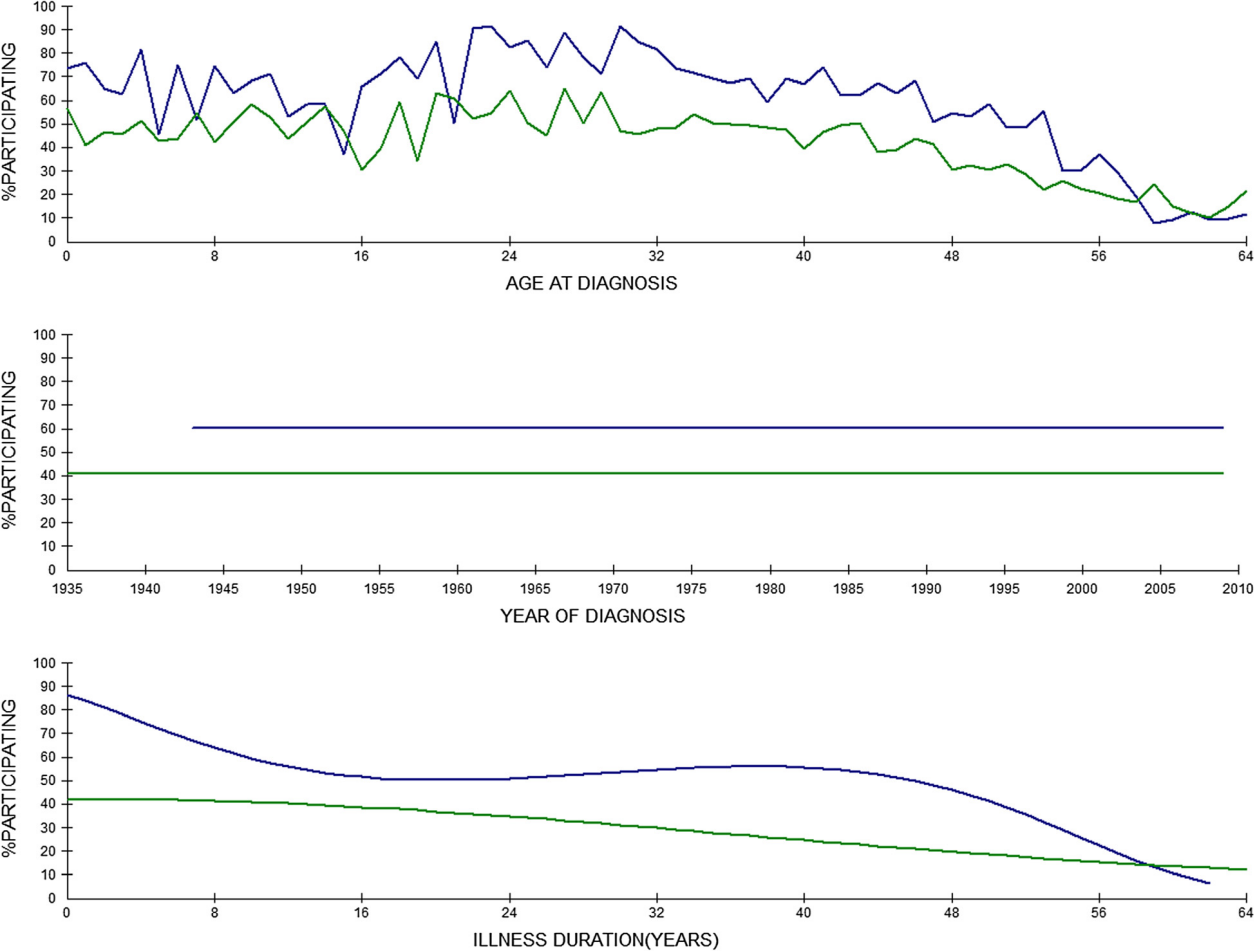
**Estimated percentages of people with chronic illness having paid work, according to their age at diagnosis, the year of diagnosis and illness duration.** Legend: Blue: men; Green: women.

Table 3 shows the effects of age at diagnosis, year of diagnosis and illness duration (all separately for men and women) on labour participation among the total sample. Model 0 shows that labour participation of both men and women with chronic illness varies according to their age at diagnosis and the year in which they had been diagnosed. Adding illness duration and several demographic characteristics to the model (model 1) results in a larger random effect of the age of diagnosis for men (not for women), whereas the random effects of the year of diagnosis almost completely disappear. Regarding the fixed part, model 1 shows that gender and the duration of a person’s chronic illness both have significant effects on labour participation chances. The effect of illness duration is not just linear and also different for men and women (χ(1) = 26.28, P < .001): for men illness duration strongly relates to their chances to participate in the labour market, whereas this is far less the case for women. Assessment of model 2 shows that, after correction for the effects of disease characteristics and the socio-economic indicators at the time of diagnosis, a person’s age at the time of diagnosis still relates to his current labour participation status. This random effect is different for men (1.56) and women (0.58) (χ(1) = 7.59, P < .01). The fixed effects of the illness duration variables for men are also still significant in this second model, but the (small) linear effect of illness duration for women has disappeared. The effects of the illness duration variables on labour participation are again very different for men and women in this model (χ(1) = 26.24, P < .001). Figure 1 shows the estimated percentages of men and women with chronic illness that participate in the labour market according to their age at diagnosis, the year of diagnosis and illness duration (number of years post-diagnosis), based on the final model.

**Table 3:** Effects of age at diagnosis and year of diagnosis (model 0), and gender and illness duration (model 1) and disease characteristics and socio-economic indicators of the year of diagnosis (model 2) on labour participation of people with chronic illness, total sample (N = 4357)

		**Model 0**^1^	**Model 1**^2^	**Model 2**^2^
		**estimate (se)**	**estimate (se)**	**estimate (se)**
**Random effects**				
- Age at diagnosis (level 2):	men	**0.99** (0.22)	**1.80** (0.37)	**1.56** (0.33)
	women	**0.86** (0.18)	**0.75** (0.16)	**0.58** (0.13)
- Year of diagnosis (level 2):	men	**0.28** (0.10)	0 (0)	0 (0)
	women	**0.27** (0.09)	0.0005 (0.02)	0 (0)
- Individual (level 1)		constraint (1)	constraint (1)	constraint (1)
**Fixed effects**				
Gender				
- Male		**0.47** (0.17)	**0.42** (0.20)	**0.41** (0.24)
- Female		**−0.57** (0.15)	**−0.36** (0.13)	−0.37 (0.20)
Illness duration				
- In years:	men		**−0.12** (0.01)	**−0.09** (0.02)
	women		**−0.05** (0.01)	−0.01 (0.02)
- In years squared:	men		**0.007** (0.001)	**0.006** (0.001)
	women		0.001 (0.001)	−0.0006 (0.001)
- In years cubed:	men		**−0.0001** (0.00002)	**−0.0001** (0.00003)
	women		−0.00002 (0.00002)	0.000005 (0.00002)
Type of chronic illness (ref. cardiovascular disease)				
- Cancer				**−0.50** (0.21)
- Diabetes				0.07 (0.15)
- Asthma / COPD				**0.54** (0.14)
- Musculoskeletal				−0.17 (0.14)
- Neurological				−0.03 (0.16)
- Digestive				0.06 (0.19)
- Other				**0.45** (0.14)
Number of chronic diseases (1–4)				**−0.40** (0.06)
Disability insurance policy in year of diagnosis (reference year: 2004)				
- Until 1966				−0.01 (0.65)
- 1967 till 1984				−0.25 (0.31)
- 1985 till 1986				0.11 (0.26)
- 1987 till 1992				−0.10 (0.15)
- 1993 till 2001				**0.0003** (0.0001)
- 2002 till 2003				**−0.45** (0.23)
- 2005				0.04 (0.27)
- 2006 till 2009				**0.51** (0.25)
Percentage registered unemployment, in year of diagnosis				−0.04 (0.02)
Gross Domestic Product – % volume change, in year of diagnosis				0.009 (0.02)

^1^model corrected for observation year.

^2^model corrected for observation year, education level, marital status, region and urbanisation level of place of residence.

in bold: significant estimates (Student’s T > 1.96, P < .05); se = standard error.

### Effects of disease characteristics and socio-economic indicators

Model 2 also shows that being diagnosed with cancer or suffering from more chronic diseases is associated with a lower chance to perform paid work, whereas being diagnosed with asthma/COPD (in comparison with being diagnosed with cardiovascular disease) relates to a higher chance to have a paid job. Furthermore, the disability insurance policy in the year of diagnosis matters for participation chances: compared to people diagnosed in 2004 (reference year), people diagnosed in 2002 or 2003 have a lower chance to participate in the labour market and people diagnosed in 2006 or later a higher chance.

### Effects for different diagnostic groups

**Figure 2:**
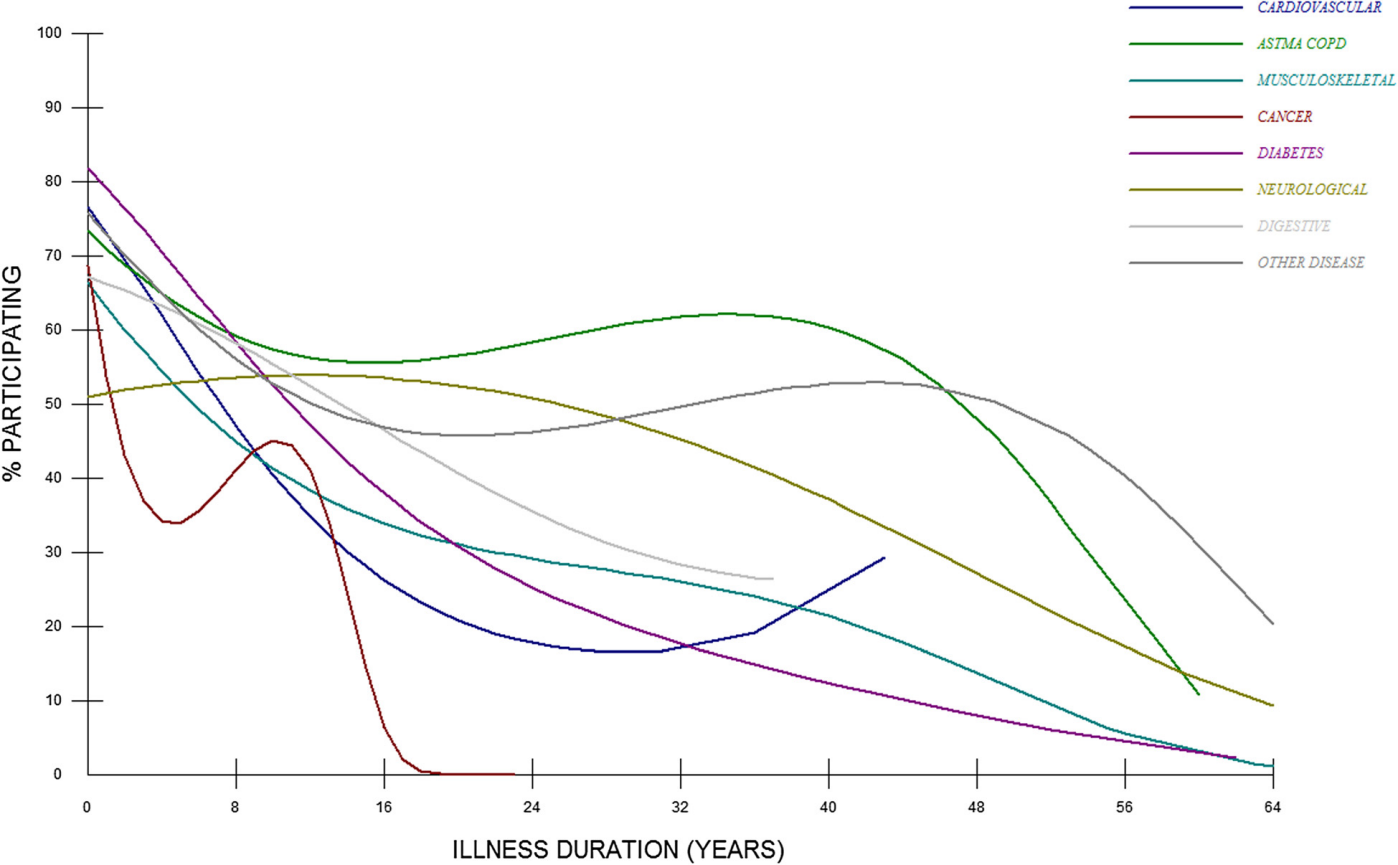
**Estimated percentages of people with different (categories of) chronic diseases, according to illness duration.** Legend: Dark blue: cardiovascular diseases; Green: asthma / COPD; Turquoise: musculoskeletal diseases; Red: cancer; Purple: diabetes; Army green: neurological diseases; Light grey: digestive diseases; Dark grey: other chronic diseases.

Table 4 contains the results of the multi-level logistic regression analysis by which we estimated the effects of the age at diagnosis, year of diagnosis and the three illness duration variables separately for each diagnostic group. This table shows that age at diagnosis accounts for a significant part of the variation in labour participation among people with cardiovascular disease, diabetes, asthma/COPD, musculoskeletal diseases and other chronic diseases, but not among the other groups. The year of diagnosis is much less important, but at least some variation in the labour participation status of cancer patients relates to the year in which they were diagnosed. Among people with cardiovascular disease or diabetes, being ill for a longer time goes along with a lower chance to participate in the labour market. In cancer patients, illness duration and labour participation are related in another way: an initial decrease of labour participation, followed by a phase in which labour participation rates increase, and finally turns again into a phase of declining participation. Figure 2 shows the estimated percentages of people with chronic illness participating in the labour market according to their illness duration (number of years post-diagnosis), based on this model.

**Table 4:** Effects of age at diagnosis, year of diagnosis and illness duration (years post-diagnosis) on labour participation status, specified for diagnostic groups, estimates and standard errors (se)

	**Cardiov.**	**Cancer**	**Diabetes**	**Asthma/ COPD**	**Musculo-skeletal**	**Neurological**	**Digestive**	**Other**
	**estimate (se)**^1^	**estimate (se)**^1^	**estimate (se)**^1^	**estimate (se)**^1^	**estimate (se)**^1^	**estimate (se)**^1^	**estimate (se)**^1^	**estimate (se)**^1^
**Random effects**								
Age at diagnosis (2)	**2.30** (1.15)	1.18 (0.75)	**2.57** (1.17)	**0.76** (0.35)	**0.81** (0.41)	0.18 (0.13)	0.00 (0.00)	**0.94** (0.44)
Year of diagnosis (2)	0.00 (0.00)	0.17 (0.25)	0.00 (0.00)	0.00 (0.00)	0.00 (0.00)	0.00 (0.00)	0.00 (0.00)	0.05 (0.06)
Individual (1)	constraint (1)	constraint (1)	constraint (1)	constraint (1)	constraint (1)	constraint (1)	constraint (1)	constraint (1)
**Fixed effects**								
Illness duration								
- In years	**−0.10** (0.03)	0.07 (0.12)	**−0.08** (0.03)	0.00003 (0.02)	−0.04 (0.02)	0.03 (0.02)	−0.03 (0.03)	−0.03 (0.02)
- In years squared	0.002 (0.003)	−0.04 (0.02)	0.001 (0.002)	0.002 (0.002)	0.002 (0.002)	−0.002 (0.002)	−0.0009 (0.004)	0.002 (0.002)
- In years cubed	−0.000002 (0.0001)	**−0.005** (0.002)	−0.00002 (0.00005)	−0.00007 (0.00004)	−0.00005 (0.00005)	0.00001 (0.00004)	0.00002 (0.0002)	−0.00004 (0.00003)

^1^model includes a constant for each chronic disease type and corrected for observation year, gender, education level, marital status, region and urbanisation level of place of residence.

In bold: significant estimates (Student’s T > 1.96, P < .05).

## Discussion

This study provides insight into the importance of three age related characteristics of people with chronic illness for their chance to participate in the labour market: illness duration (years post-diagnosis), the age at the moment of diagnosis and the year in which the diagnosis was established.

### Factors associated with labour participation status

#### Illness duration

As we hypothesized, people with a longer illness duration are less likely to participate in the labour market. This may be explained by the fact that most chronic diseases are progressive in nature, resulting in more functional limitations over time. The small, but significant positive relationship between illness duration and severity of the chronic condition supports this explanation. The development of comorbidities might also play a role in this respect, since a longer illness duration (calculated from the first chronic disease) is also related to the total number of chronic diseases diagnosed in a person. However, including the number of chronic diseases as an independent variable in our model (Table 3, model 2) showed a negative effect of its own, indicating that multi-morbidity in itself is associated with a lower chance of labour participation in addition to illness duration.

The linear relationship between illness duration and labour participation is much stronger for men than for women. Furthermore, the effect of illness duration on the labour participation status of chronically ill men is not just linear. The stronger illness duration effects for men suggest that the duration of chronic illness is more decisive for the labour participation status of men than of women. Though this does not correspond with the often reported higher elasticity of labour supply of women compared to men, it is in line with other studies demonstrating that labour participation of women is less sensitive to health problems [8]. Other factors (e.g. being married, raising children) may influence women’s decision to continue or stop working as well [14], thereby reducing the importance of health problems. Labour participation of (also healthy) women in the Netherlands is still relatively low compared to Northern and most Western European countries and the majority of Dutch women who are employed work part-time [46]. The diagnosis-specific analysis showed that how illness duration relates to labour participation also depends on the type of chronic disease at stake: linear (negative) effects were found among people with cardiovascular disease or diabetes, whereas a more fluctuating pattern was found among cancer patients.

#### Age at diagnosis

Also in line with our hypotheses, we found that the age at diagnosis is important for labour participation chances later in life. Again, this holds more for men than for women. Since we included the ages at diagnosis in the random part of our models, we could not specify the nature of the relationship. The diagnosis-specific analysis suggests that the significance of diagnostic age for labour participation in later life depends on the type of chronic disease at stake. In this respect we wish to mention that the chronic diseases of our respondents differed from each other in many ways including the age (range) at which they are usually diagnosed.

#### Year of diagnosis

We did not find evidence for the hypothesis that variation in labour participation among people with chronic illness relates to the year of diagnosis. The significant effects of the year of diagnosis we found in an initial model disappeared when the illness duration of the respondents and some demographic characteristics were taken into account. However, concluding that the historical time period in which the diagnosis of a chronic disease was established is irrelevant for employment chances later in life seems premature, since only a small part of our sample had been diagnosed in early years (< 10% before 1980). Moreover, at least some variation in labour participation of cancer patients relates to the year in which they had been diagnosed. Thus, it is still possible that this is indeed a relevant factor in cancer survivors or other diagnostic groups.

#### Other factors

Apart from their illness duration and age at diagnosis, labour participation of people with chronic illness also relates to the type of chronic disease and how many chronic diseases they suffer from. Furthermore, our findings provide some indication that the gradually more restrictive disability insurance policy of the Netherlands has resulted in more chronically ill people remaining economically active, especially since 2006 when a new law (see note b) emphasizing reintegration instead of income protection was introduced.

### Methodological considerations

One might argue that the effects of illness duration and age at diagnosis are nothing else than the reflection of an overall age effect. The age of the respondent is indeed similar to his age at diagnosis plus his illness duration. However, by distinguishing these two components (and assessing them simultaneously by our multi-level approach) we were able to provide more detailed information. The relevance of our approach is demonstrated by the different effects these variables have on labour participation of different diagnostic groups. While the single-level logistic regression analyses showed that age (at the time of measurement) negatively relates to labour participation within all diagnostic groups, the multi-level analysis demonstrated that age at diagnosis does not always account for a significant part of the variation in labour participation status among all diagnostic groups, nor is illness duration always significantly related.

Our results do not provide much insight into the specific labour situation of people with neurological diseases or digestive disorders. This may be due to the heterogeneity of these groups with regard to the course of illness and nature/burden of disease. Heterogeneity in this respect will to some extent be present in all diagnostic groups we included in this study. Unfortunately our sample was too small to distinguish specific chronic diseases such as heart failure or breast cancer. Samples allowing researchers to be more precise regarding the type of chronic disease could provide more insight.

Another point of discussion is the fact that our sample naturally consisted of chronic patients who could be considered as survivors. The retrospective nature of the study implies that, strictly speaking, we cannot conclude that the age at diagnosis relates to the labour participation status of people with chronic illness later in life, since not all people diagnosed with a chronic disease were represented by our sample at the time of measurement (e.g. people who were already too ill to participate in the panel or who had passed away).

### Implications for policy and practice

We believe that our results provide some insights that can be used for policy and practice. First, our findings show that within a sample of people who all suffer from chronic illness, there is still much variety in labour participation status. This does not only relate to the type of chronic disease these people are diagnosed with, but also to how old they were when chronic illness came into their lives and how long they have already lived with their illness. Since a longer illness duration generally relates to a smaller chance of labour participation, (long-term) sick-leave during the first year(s) of chronic illness should be prevented as much as possible, since successful reintegration is less likely to occur with prolonged illness. Furthermore, knowing that a chronic disease is at stake, companies and their medical officers should not only focus on return to work, but also support their chronically ill employees to adapt to working with a chronic illness. This asks for proactive management: anticipating on future health deterioration (for most chronic diseases) and taking appropriate measures to prevent drop-out. Third, also within general healthcare attention should be paid to working with chronic illness. Since the age at diagnosis does matter for current and future employment chances of people with chronic diseases, addressing age (and gender) specific career issues should be part of chronic disease management right from the diagnosis.

## Conclusions

We conclude that differences in labour participation of people with chronic illness partly relate to the age they had when they were diagnosed. This especially applies to men and to people diagnosed with major chronic diseases such as diabetes, cardiovascular disease, asthma/COPD, or musculoskeletal diseases. A longer illness duration generally relates to a lower chance of people with chronic illness to be economically active, but for cancer patients/survivors it depends more on the phase of the illness they find themselves in. Prospective studies are needed to assess illness trajectories of specific diagnostic groups along with the development of their school and work careers.

### Endnotes

^a^These conditions are: 1. the data will be used for research purposes that fit in with the general purpose of the panel-study (contribute to scientific knowledge on how to improve quality of care, quality of life and participation from the patient perspective), 2. the results of the study will be published in a public report or paper, 3. the study plan does not overlap with studies that are already carried out with the same data or have already been planned, 4. the concept publication is sent to the project leader (MR) to check the correct use of the data, 5. additional costs for preparation of the data set need to be paid.

^b^Members of the steering committee are representatives of the Dutch Council of the Chronically ill and the Disabled (a national umbrella organization consisting of associations of people with a chronic illness or a disability), the Netherlands ministry of Health, Welfare and Sports, the Netherlands ministry of Social Affairs and Employment and the Netherlands Institute for Social Research.

^c^We did not collect data about the number of hours worked at the first measuring moment of each cohort. Therefore, we could not specify the variable labour participation.

^d^Until 1967 there were only very limited social security arrangements for employees who became occupationally disabled. In 1967 the Disability Insurance Act was introduced; eligibility criteria were rather broad and benefits were relatively high. In 1985 benefits were decreased to reduce expenditures. In 1987 the eligibility criterion was changed: individuals who were considered partially disabled from a medical point of view were no longer treated as fully occupationally disabled if it was assumed they could find a job. From 1993 on several measures have gradually been taken to restrict the inflow and to re-examine beneficiaries (partly by using more restrictive criteria). In 2006, the Disability Insurance Act was replaced by the ‘Work and Income according to Labour Capacity Act’, which puts a high emphasis on reintegration and provides benefits only for those who have a labour capacity loss (based on the capacity to do any kind of work, whether or not available) of more than 35 percent. The new law can be considered the most restrictive disability legislation since 1967.

## Competing interests

The authors declare that they have no competing interests.

## Authors’ contributions

MR and PS designed the study. MR was responsible for the data collection in the National Panel of People with Chronic illness or Disability (NPCD) and wrote all drafts of the manuscript. MR and PS conducted the statistical analyses (MR performed the descriptive analyses and the single-level logistic regression analyses; PS designed and performed all multi-level analyses). JS and PPG provided theoretical and empirical input to the study design and the manuscript, and commented on all drafts of the manuscript. PPG also contributed to the statistical analysis plan. All authors read and approved the final manuscript.

## Pre-publication history

The pre-publication history for this paper can be accessed here:

http://www.biomedcentral.com/1471-2458/13/803/prepub
